# The Influence of Tanning Chemical Agents on DNA Degradation: A Robust Procedure for the Analysis of Tanned Animal Hide—A Pilot Study

**DOI:** 10.3390/life14010147

**Published:** 2024-01-19

**Authors:** Kristyna Hebenstreitova, Ondrej Salaba, Jakub Trubac, Jitka Kufnerova, Daniel Vanek

**Affiliations:** 1 Institute for Environmental Sciences, Charles University, Benátská 2, 128 00 Prague, Czech Republic; 2 Faculty of Forestry and Wood Sciences, Czech University of Life Sciences, Kamýcká 129, 165 00 Prague, Czech Republic; 3 Institute of Geochemistry, Mineralogy and Mineral Resources, Faculty of Science, Charles University, Albertov 6, 128 00 Prague, Czech Republic; 4 CRL Radiocarbon Laboratory, Department of Radiation Dosimetry, Nuclear Physic Institute of the Czech Academy of Sciences, Na Truhlářce 38, 180 86 Prague, Czech Republic; 5 Forensic DNA Service, Budínova 2, 180 81 Prague, Czech Republic; 6 Bulovka University Hospital, Budínova 2, 180 81 Prague, Czech Republic; 7 2nd Faculty of Medicine, Charles University, V Úvalu 84, 150 00 Prague, Czech Republic

**Keywords:** wildlife crime, degradation index, quantification, tanning

## Abstract

Illegal wildlife trade is currently on the rise, and it is becoming one of the most lucrative crime sectors. The rarer the species, the higher the demand. Wildlife trade falls under international regulations, such as the CITES convention. Proving that this convention has been violated is a complex process and can be very difficult to do. DNA analysis methods remain (in many cases) the only way to determine whether a certain specimen originated from a protected animal species, a specific individual, or a species in which it is legal to trade. Tanned animal hides are a specific type of specimen. With this type of biological material, obtaining amplifiable DNA is often difficult. This pilot study aimed to map the effect of the chemicals used in the tanning process on the degradation of the DNA yielded from such samples. The DNA was quantified using two different approaches: qPCR and Qubit fluorometry. The degree of DNA fragmentation was assessed by determining the degradation index. The results indicate that reagents containing chromium have the greatest influence on DNA degradation. However, by using the presented protocol, enough amplifiable DNA can be obtained from hides treated with aluminum-based reagents.

## 1. Introduction

Commerce with wild animal species, whether lawful or unlawful, has catastrophic implications for the environment. In addition to devastating the natural habitats of wild animals, commerce in wild animals stands out as one of the foremost factors imperiling the survival of numerous types of wildlife [1,2]. The trade of endangered wild animals is governed by the international agreement known as CITES (Convention on International Trade in Endangered Species of Wild Fauna and Flora), which was established in 1973. At present, the convention encompasses a staggering array of over 35,000 animal and plant species, classified into appendices based on the extent of their vulnerability. Appendix I encompasses species that face direct threats of extinction, resulting in a complete global prohibition on their trade. Appendix II comprises species that may become endangered if their trade remains unregulated. Appendix III incorporates species protected in at least one country that has sought assistance from other CITES signatory parties to regulate the trade of said species [3].

Illegal trade in endangered animal species ranks as one of the most profitable organized crime sectors, following the narcotics trade, human trafficking, and counterfeit goods in terms of global profit [4,5]. The illegal trading of wild animals involves poaching, smuggling, capturing, and breeding endangered and protected animal species for various purposes [6]. One of these purposes includes the trade of various animal-derived products, such as teeth, claws, or hides, which are highly sought after on the black market. The hides of animals from the genus *Panthera*, which includes many endangered species, are particularly valuable [7,8,9,10,11].

To facilitate the illicit transportation of animal hides and maintain their value, it is essential to subject them to a protective treatment process—the hides must undergo tanning. Tanning represents one of the earliest preservation methods developed by humans, enabling the retention and manipulation of animal hides as a versatile resource. The versatility of tanning is so extensive that it can be applied in both domestic and industrial settings [12,13]. Regrettably, this reality is exploited by individuals involved in the unlawful trafficking of endangered animal species.

Lutan FN, Novaltan AL, and chromium sulfate are, according to taxidermists and tanners, currently among the most commonly used tanning agents. Therefore, it is essential to assess their impact on DNA integrity and their subsequent suitability for downstream analyses. Presumably, chromium sulfate has the most devastating effect on DNA integrity [14].

It is estimated that approximately 70% of criminal offenses associated with wildlife trafficking fail to result in legal action due to the inaccurate identification of the species from confiscated animal materials [15]. Accurate species identification is of paramount importance in investigations, as it is the key factor in determining whether the seized materials are derived from endangered animal species. In certain forensic cases, a DNA analysis can serve as the sole method for establishing species affiliation [5,15].

Biological artifacts from endangered species are becoming more prevalent among forensic samples. The illicit trafficking of body parts and goods derived from individuals of the subfamily *Pantherinae*, commonly known as big cats, is widespread not only in Asia but also in Europe. This can be attributed in part to the substantial demand from Asian communities residing outside Asia [8]. Tanned hides are one of the frequently traded commodities on the black market [7].

Tanned hides represent a specific category of biological material that poses challenges for obtaining viable DNA, with DNA extracted from such material frequently being heavily degraded [16]. It is hypothesized that the chemicals employed in the tanning process directly contribute to DNA degradation [17]. Hence, the development of a robust procedure is imperative for handling forensic samples derived from tanned hides, allowing for a DNA analysis and the subsequent identification of species or individuals.

## 2. Materials and Methods

### 2.1. Materials

A *Panthera pardus* hide was tanned and used for all subsequent analyses in this study. The hide was obtained from a deceased individual and was kindly provided by Jihlava Zoo. For the sampling that was conducted at various stages of tanning, a labeling method was established (Table 1 legend). All the samples were labeled with the letter “L”, reflecting their biological origin (leopard skin). The tanning process was divided into nine stages, and a hide sample was taken from each individual stage. The specific stage is indicated by a number in the sample label (e.g., the third tanning stage—pickling—is labeled L3). Three different tanning agents were used individually on separate samples in the tanning process: Lutan FN, Novaltan AL, and a chromium sulfate agent (all from Bauer Handels, Fehraltorf, Switzerland). The following abbreviations were assigned to the samples treated with each specific agent: Lutan FN (-L), Novaltan AL (-N), and the chromium sulfate agent (-C). Finally, it should be noted that stages L1–L5 denote the early preparatory stages; therefore, the samples treated with the three agents were prepared for tanning treatment in the same manner. The specific tanning agent was introduced into the tanning process at stage L6, where the process stages were divided into three substages depending on the agent used.

### 2.2. Tanning

For the tanning of the hide, several solutions were used (Table 2). All the solutions were freshly prepared and used only for tanning the studied *Panthera pardus* hide.

Stage L1

Samples taken from this stage represent the untreated hide, which was only cleaned of fat and tendons.

Stage L2

In the second tanning stage, a process called “soaking” was performed. The hide was immersed in a degreasing solution for 2 h. Then, the hide was transferred to a new solution with an exposure time of 30 min. Subsequently, the hide was rinsed in clean water at a temperature of 30 °C for 5 min. Finally, the hide was centrifuged. At this stage, impurities and globular proteins were removed, and the hide was degreased.

Stage L3

In the third tanning stage, the hide was transferred to pickling solution I. The exposure time was 48 h, and the solution pH was 1.5. After the pickling process, the pH of the solution was 3. At this stage, the hide was acidified to enhance the binding of the tanning agent molecules to the carboxyl groups of collagen.

Stage L4

In the fourth tanning stage, the hide was thinned using a circular fleshing machine while preserving the epidermis and an approximately 3 mm thick portion of the dermis. This was followed by a degreasing soak, in which the hide was immersed for 15 min, rinsed with lukewarm water at a maximum temperature of 35 °C, and then centrifuged. Notably, the higher-temperature soaking allowed for better degreasing but then had to be followed by the reacidification of the hide in a subsequent pickle, as will be discussed.

Stage L5

In the fifth tanning stage, the hide was transferred to pickling solution II. The exposure time was 5 h, and the solution pH was 2.5. After the pickling process, the hide was allowed to drain for two hours and transferred to stage L6. Stage L5 had to be included to reacidify the skin to pH 3.5 to 4.0 for the better binding of tannins (tanning agent) in the stage of L6 tanning itself. After drying, the hide pH was 4. At this stage, the hide was reacidified before the actual tanning process.

Stage L6

In the sixth stage, the actual tanning of the hide was performed, along with the addition of fat emulsions. The hide was divided into three parallel pieces (L6-L, L6-N, and L6-C). Each piece was treated with a different agent (Lutan FN, Novaltan AL, or chromium sulfate agent). This and all subsequent tanning stages were performed separately for each piece. The hide pieces were immersed in their respective solutions for 48 h. The solution pH was 3.5 to 4.5 for the Lutan FN and Novaltan AL tans. For chromium sulfate tanning, the solution pH was 3.5 to 4.0. The pH of the making solutions was changed during the process. This led to a softening of the structure of the tanned skins, the removal of residual fat, and the optimization of skin moisture.

Stage L7

In the seventh stage, Eulan SPA 01 was added to each tanning solution to provide protection against insects. The exposure time was 2 h. Hides tanned in this manner are commonly used for taxidermy (after fleshing), and further greasing and drying are not performed. The moist hides are instead stretched onto a model. However, for our purposes, additional finishing stages (phases L8 and L9) were performed.

Stage L8

In the eighth stage, the hide pieces were treated with a mixture composed of Prinol M 31, Pelgrasol SF, and water at a temperature of 50 °C. The treatment was applied to the side of the hide without fur. The hide pieces were incubated for 2 h at room temperature before further processing.

Stage L9

In the ninth stage, the hide pieces were softened through a process called “tumbling” or “milling.” This process involved placing the hides in a special drum with the addition of sawdust from deciduous trees and perchloroethylene. This led to a softening of the structure of the tanned hides, the removal of residual fat, and the moistening of the hides.

After phase L9, the hides were stretched and dried on a frame.

### 2.3. Sampling

To ensure the standardization of the input material for subsequent analyses, skin punch biopsy specimens with a diameter of 2.5 mm were obtained from the hide pieces using RP03 rapid leather punch pliers set at the smallest configuration.

### 2.4. DNA Extraction

To investigate the specific nature of the samples, we optimized a protocol for DNA extraction from solid tissues using a Quick-DNA Miniprep Plus Kit (Zymo Research, Irvine, CA, USA). The protocol was modified as follows:

- Each hide sample obtained by biopsy was placed into a 2 mL ZR BashingBead Lysis Tube (Zymo Research, USA) filled with 600 µL of DNA/RNA Shield (Zymo Research, USA).
- The lysis tubes were then placed in a homogenizer (Disruptor Genie, Scientific Industries, Bohemia, NY, USA), and the mixture was homogenized for 6–10 min.
- After homogenization was completed, the tubes were briefly centrifuged (for approximately 10 s) at room temperature.
- Then, 300 μL of lysis buffer (Solid Tissue Buffer, Zymo Research), 20 μL of proteinase K, and 20 μL of dithiothreitol (DTT) were added.
- The mixture was vortexed for 10–15 s and incubated at 55 °C overnight.
- After the incubation was completed, centrifugation was performed for 1 min at 10,000 g and room temperature.
- The supernatant was transferred to a new 2 mL microcentrifuge tube.

After this last step, the unmodified Quick-DNA Miniprep Plus Kit (Zymo Research) manufacturer’s protocol was followed, with an elution volume of 50 μL. All samples were subsequently purified using a OneStep PCR Inhibitor Removal kit (Zymo Research).

DNA was extracted from the hide pieces obtained by punch biopsy from a total of 17 samples, labeled L1 to L9, as described in previous sections. The DNA extraction process was carried out in duplicate (two parallel extractions).

### 2.5. DNA Quantification

#### 2.5.1. qPCR

For DNA quantification and the calculation of the degradation index, two pairs of primers targeting housekeeping genes were selected (Table 3) [18,19]. The optimal annealing temperature was subsequently tested using gradient PCR. The Basic Local Alignment Search Tool (BLAST) (Bethesda, MD, USA) on the NCBI website was used to ensure that the primers targeted the DNA of *Panthera pardus*.

The DNA was quantified using the SYBR Green method. A standard curve was designed using *Panthera pardus* DNA of a known quantity at concentrations of 2 ng/µL, 0.2 ng/µL, 0.02 ng/µL, and 0.002 ng/µL (133 bp target), and 0.4 ng/µL, 0.04 ng/µL, 0.004 ng/µL, and 0.0004 ng/µL (369 bp target). The qPCR Master Mix and amplification protocol are described in Table 4 and Table 5, respectively. All samples were analyzed in technical triplicate. Data were analyzed using QuantStudio Design and Analysis Software v 1.5.2 (Thermo Fisher Scientific, Waltham, MA, USA).

#### 2.5.2. Fluorometry

The DNA was also quantified using a Qubit 4 Fluorometer (Thermo Fisher Scientific) with a dsDNA HS Assay Kit, following the manufacturer’s protocol.

### 2.6. Calculation of the Degradation Index

The quantification data derived from the signal of small amplicons in qPCR were compared with the data obtained from the large amplicon signal to evaluate the extent of DNA degradation in the samples. Given that larger DNA fragments are more prone to degradation than smaller fragments, the ratio between the signals of the two probes can be utilized to calculate the degradation index (DI) using the following mathematical formula:

$$DI=\frac{Concentration\;of\;small\;DNA\;fragment}{Concentation\;of\;large\;DNA\;fragment\;}$$

Through the calculated DI, the degree of DNA degradation can be assessed, enabling the evaluation of its appropriateness for subsequent analyses [20].

### 2.7. DNA Typing

For the determination of the leopard’s DNA profile, the identification system Ptig STRPlex was utilized [9], and the protocol described in [9] was followed. The DNA extracted from the hide sampled at the beginning and end of the tanning process (phases L1 and L9) was used for the analysis.

### 2.8. XRF

The elemental composition of the tanned hide was determined using X-ray fluorescence spectrometry (XRF), a noninvasive in situ method. The L9-L, L9-N, and L9-C samples were measured using a portable XRF analyzer (Niton XL3t XRF Analyzer, Thermo Fisher Scientific, USA). This analysis eliminates the need for sample preparation and enables measurements to be taken under ambient air conditions. As a result, a rapid preliminary spot analysis at various locations on the hide was possible.

## 3. Results

### 3.1. DNA Quantification

DNA was successfully quantified in all samples analyzed. Overall, the DNA remained amplifiable throughout all the samples taken from all the tanning stages.

**Figure 1:**
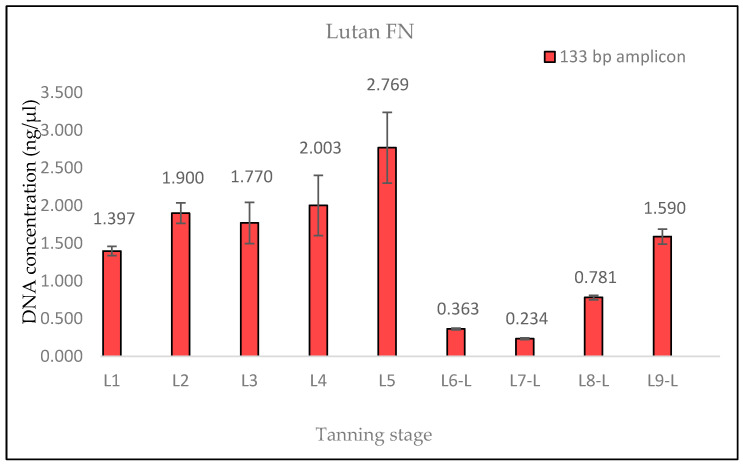
Histograms showing the qPCR quantification of 133 bp amplicon results for the Lutan FN-tanned samples. DNA extracted from hide samples at different stages of tanning (*x*-axis) was used as template DNA. DNA concentration values in ng/μL (*y*-axis) for each sample are shown as the mean of the measurements and their standard deviations. The measured 1DNA concentration values in samples L8-L and L9-L did not follow the expected trend.

**Figure 2:**
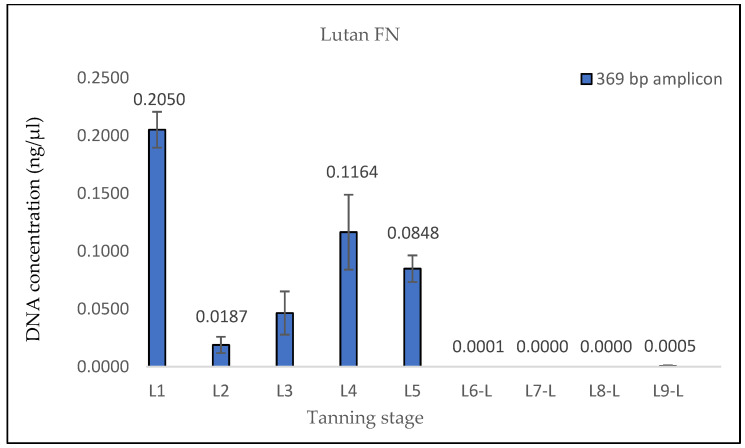
Histograms showing the qPCR quantification of 369 bp amplicon results for Lutan FN-tanned samples. DNA extracted from hide samples at different stages of tanning (*x*-axis) was used as template DNA. DNA concentration values in ng/μL (*y*-axis) for each sample are shown as the mean of the measurements and their standard deviations. The measured DNA concentrations values were very low after the initiation of the actual tanning (L6 phase), indicating that the DNA was highly degraded in these samples.

**Figure 3:**
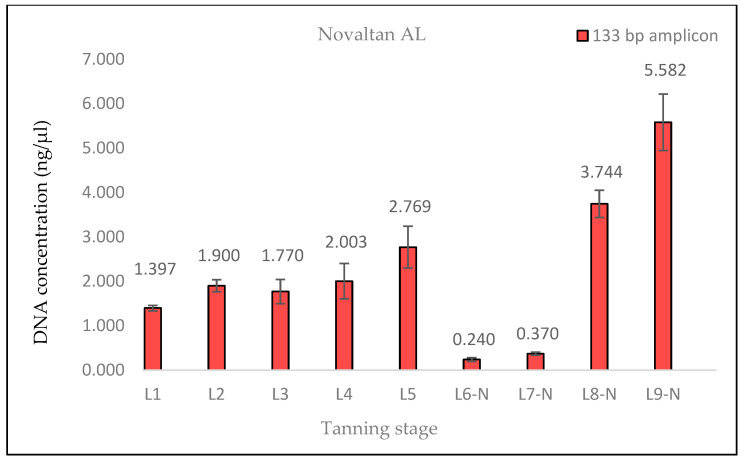
Histograms showing the qPCR quantification of 133 bp amplicon results for Novaltan AL-tanned samples. DNA extracted from hide samples at different stages of tanning (*x*-axis) was used as template DNA. DNA concentration values in ng/μL (*y*-axis) for each sample are shown as the mean of the measurements and their standard deviations. The measured DNA concentration values did not follow the expected trend.

**Figure 4:**
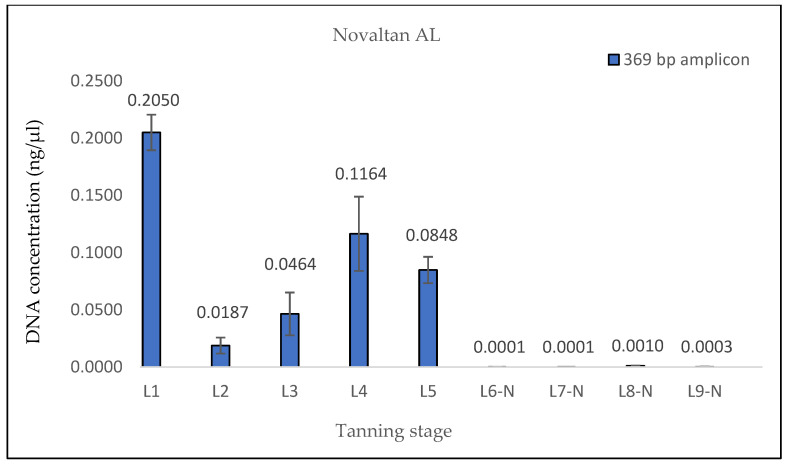
Histograms showing the qPCR quantification of 369 bp amplicon results for Novaltan AL-tanned samples. DNA extracted from hide samples at different stages of tanning (*x*-axis) was used as template DNA. DNA concentration values in ng/μL (*y*-axis) for each sample are shown as the mean of the measurements and their standard deviations. The measured DNA concentrations values were very low after the initiation of the actual tanning (L6 phase), indicating that the DNA was highly degraded in these samples.

The measured DNA concentrations of the 133 bp amplicon for samples L1 and L2 exhibited relative similarity; however, the degree of DNA degradation was notably higher in the L2 sample. In the case of sample L3, an increase in the DNA concentration of the 369 bp amplicon and, therefore, a decrease in the degree of DNA degradation were observed. This consistent increase was evident even in quantifications conducted prior to the optimization of the DNA extraction protocol, suggesting that it was not a random occurrence (see Discussion). Subsequently, in samples L4 and L5, the degree of DNA degradation did not follow the expected trend, as it decreased. A significant decline in the DNA concentration of the 369 bp amplicon was observed from the L6 pieces onward, aligning with the point at which the hides came into contact with the three different tanning agents. In stages L8 and L9, an unexpected increase in the DNA concentration of the 133 bp fragment, in both samples treated with Lutan FN and Novaltan AL tanning agents (L8-L, L8-N, L9-L, and L9-N), was observed (Figure 1, Figure 2, Figure 3 and Figure 4). The same results, which are contrary to the anticipated trend, are seen in DNA quantification using a Qubit Fluorometer. This suggests that it is not a phenomenon coupled only with qPCR quantification. However, there was no corresponding increase in the measured concentrations of the longer DNA fragment in these samples.

**Figure 5:**
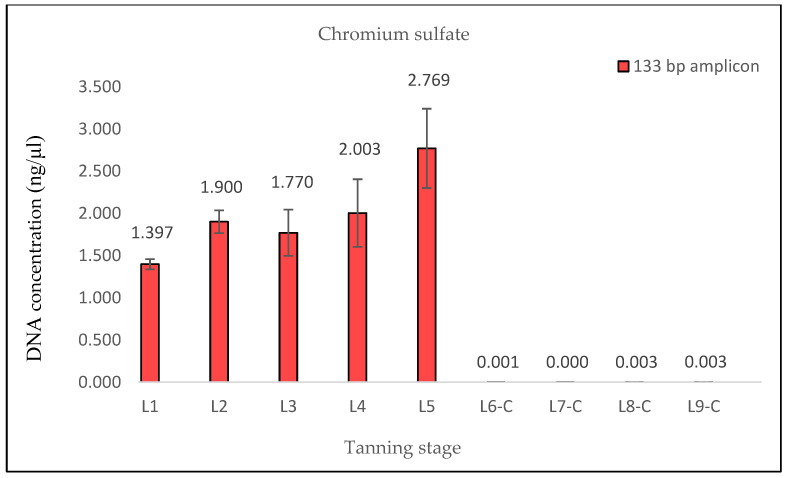
Histograms showing the qPCR quantification of 133 bp amplicon results for chromium sulfate-tanned samples. DNA extracted from hide samples at different stages of tanning (*x*-axis) was used as template DNA. DNA concentration values in ng/μL (*y*-axis) for each sample are shown as the mean of the measurements and their standard deviations. DNA concentrations values were very low after the initiation of the actual tanning (L6 phase), indicating that the DNA was highly degraded in these samples.

**Figure 6:**
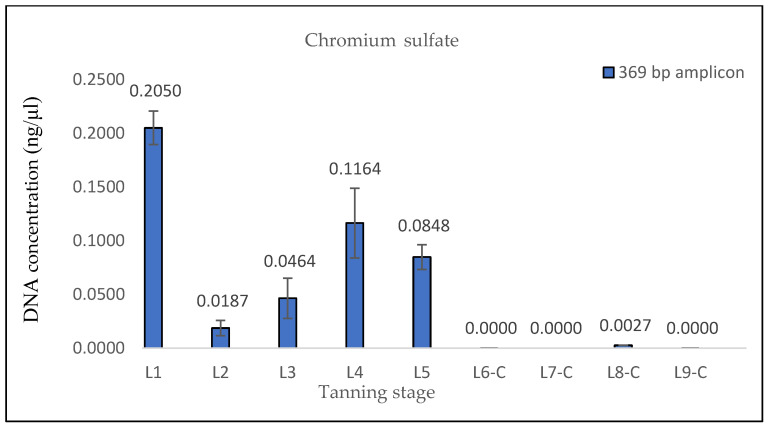
Histograms showing the qPCR quantification of 369 bp amplicon results for chromium sulfate-tanned samples. DNA extracted from hide samples at different stages of tanning (*x*-axis) was used as template DNA. DNA concentration values in ng/μL (*y*-axis) for each sample are shown as the mean of the measurements and their standard deviations. In samples obtained after the initiation of the actual tanning step (L6 phase), no notable amplification of the 369 bp amplicon was observed, suggesting that only fragments < 369 bp are likely to be present in these samples and that the DNA is therefore highly degraded.

The samples L6-C, L7-C, L8-C, and L9-C did not exhibit any notable amplification of the longer DNA fragment (369 bp), indicating that the chromium sulfate tanning agent induced the most substantial DNA degradation, which is consistent with the hypothesis (Figure 5 and Figure 6).

The degree of DNA fragmentation, which reflects how much DNA has been degraded during hide tanning, was determined using DI (Table 6). The determination of DI was not possible for samples L6-C, L7-C, L8-C, and L9-C due to the absence of amplification for the longer fragment (369 bp) in qPCR. This implies that the DNA in these samples was degraded into fragments smaller than 369 bp, indicating that the chromium sulfate tanning process resulted in the most significant DNA degradation. It can be concluded that all evaluated samples, except for sample L1, exhibited substantial DNA degradation (DI > 10).

**Figure 7:**
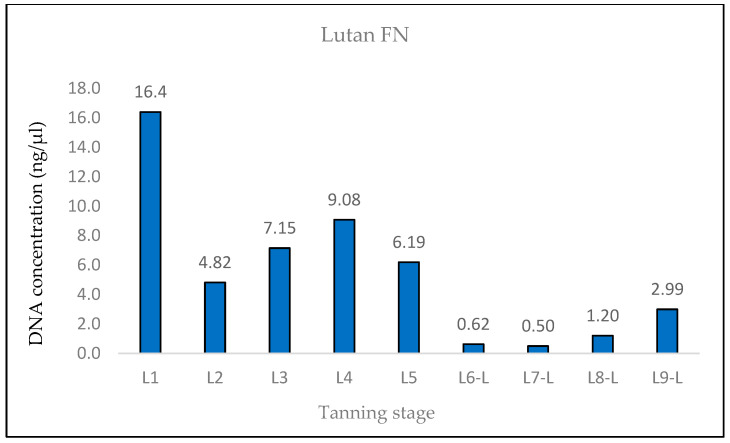
Histogram showing the results of quantification of genomic DNA extracted from Lutan FN-tanned hides at different stages of tanning by fluorometric measurement on a Qubit system. A rapid decrease in DNA concentrations was observed between the L5 and L6 phases, which is consistent with the expected trend. The values of DNA concentrations measured in samples L8-L and L9-L are outside the expected trend.

**Figure 8:**
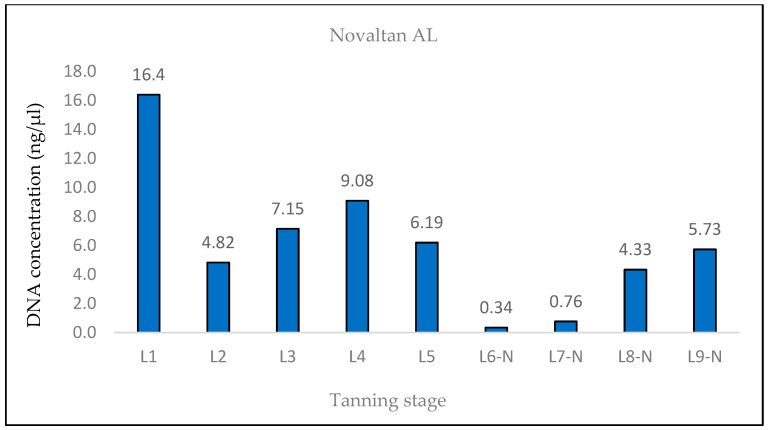
Histogram showing the results of quantification of genomic DNA extracted from Novaltan AL-tanned hides at different stages of tanning by fluorometric measurement on a Qubit system. A rapid decrease in DNA concentrations was observed between the L5 and L6 phases, which is consistent with the expected trend. The values of DNA concentrations measured in samples L8-N and L9-N are completely outside the expected trend.

**Figure 9:**
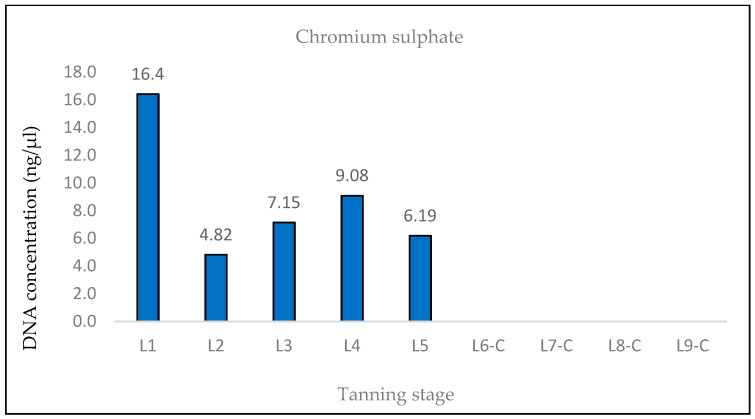
Histogram showing the results of quantification of genomic DNA extracted from chromium sulfate-tanned hides at different stages of tanning by fluorometric measurement on a Qubit system. A rapid decrease in DNA concentrations was observed between the L5 and L6 phases, which was consistent with the expected trend. For samples L6-C, L7-C, L8-C, and L9-C, the DNA concentration was below the detection value of the system (<100 pg/μL).

The DNA was also quantified using the Qubit system. The expected trend of decreasing DNA concentration throughout the tanning process was observed (Figure 7, Figure 8 and Figure 9). In the case of samples treated with Lutal FN and Novaltan AL, the L8-N and L9-N samples displayed an elevated DNA concentration, contrary to our assumption. For samples L6-C, L7-C, L8-C, and L9-C, the total DNA concentration fell below the detection threshold of the system (<100 pg/μL), resulting in no measurable value (Figure 9).

### 3.2. DNA Typing

An STR analysis was conducted to establish the individual DNA profiles of the following samples: L1, L9-L, L9-N, and L9-C. Sample L1 served as the reference to assess the comprehensiveness of the DNA profiles obtained (Figure S1). Due to its origin being an untanned hide, it was considered suitable for obtaining a complete DNA profile. Samples L9-L, L9-N, and L9-C were selected to assess the feasibility of determining a DNA profile from fully tanned hide samples.

A complete DNA profile was successfully obtained from all the analyzed samples, except for sample L9-C, where only a partial DNA profile could be obtained (Figures S1–S8).

The L9-L sample exhibited an electrophoretic artifact known as the “pull-up” effect, characterized by excessive fluorescence detected in one color channel that spreads into other channels, appearing as a distinct peak (Figures S3 and S5). This artifact was likely caused by the compromised amplification of longer DNA fragments and the preferential amplification of shorter fragments due to DNA degradation in the L9-L sample.

All obtained DNA profiles are available as part of the Supplementary Materials.

### 3.3. Chromium Detection in Hide Samples

**Figure 10:**
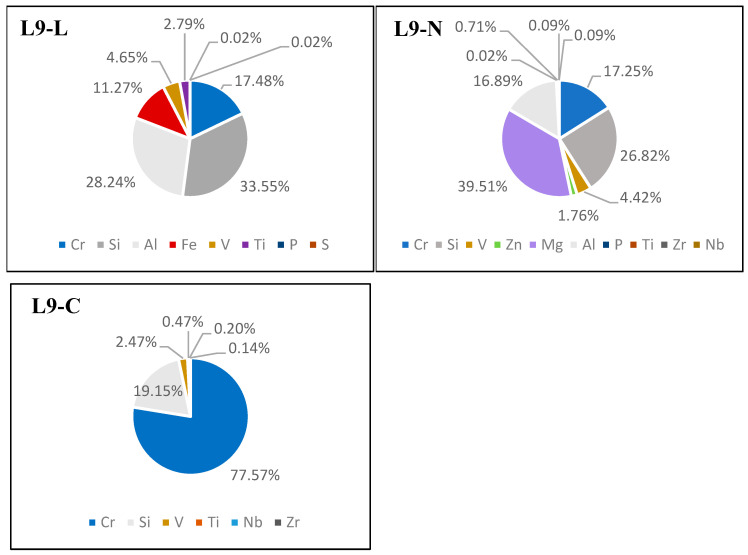
Elemental composition of the tanned hides measured via XRF.

The measurements revealed the presence of chromium in all three tanned leather samples (L9-L, L9-N, and L9-C). As anticipated, the L9-C sample exhibited the highest concentration of chromium. Additionally, the composition of other elements in the leather was also determined (Figure 10).

## 4. Discussion

Tanned hides are a problematic type of biological material in terms of genetic analysis, as the tanning process has a very negative effect on DNA integrity [17,21]. Another obstacle to successful genetic analysis arises from the PCR inhibitors that are copurified with DNA during the isolation process. A previous study conducted in 2007 exploring the impact of vegetable tanning on DNA amplification in PCR demonstrated that only mtDNA amplification was successful, while nuclear DNA amplification was not [16]. The authors attributed this outcome to DNA degradation during tanning and the presence of PCR inhibitors. In contrast, our study achieved the amplification of nuclear DNA in all the examined samples. However, this favorable outcome is likely attributable to the improved sensitivity of the PCR-based methods currently employed, in line with our findings.

Our findings indicate that the various tanning methods have distinct impacts on DNA integrity. However, based on our results, we infer that many substances employed in the tanning process exert a detrimental influence on the structure of DNA. This observation aligns with the conclusions drawn in previous studies conducted by Vuissoz et al. [16] and Ražić et al. [17].

To overcome the challenges of obtaining subamplifiable DNA yields and the copurification of PCR inhibitors during the DNA extraction process, we optimized the protocol for DNA isolation from solid tissues using a commercially available kit commonly utilized in our laboratory (Quick-DNA Miniprep Plus Kit, Zymo Research). Subsequently, we validated this optimized protocol specifically for the extraction of DNA from tanned hides.

The low DNA yields obtained from tanned hides may be attributed to the inherent nature of the tanning process, particularly the formation of crosslinking between collagen fibers [22]. However, importantly, such crosslinking can also occur between collagen and DNA, making it challenging to effectively release DNA from this structure during the extraction process. Therefore, our focus was primarily on optimizing the lysing step within the extraction process.

To achieve efficient lysis, a combination of mechanical lysis using specialized lysis tubes (BashingBead Lysis Tubes, Zymo Research) and chemical lysis using the lysis buffer provided by the manufacturer, proteinase K, and DTT were employed. To ensure the maximum preservation of DNA integrity during sample homogenization within the lysis tubes, the tubes were filled with a protective buffer (DNA/RNA Shield, Zymo Research). The utilization of lysis tubes has previously demonstrated increased DNA yields [23], while the DNA/RNA Shield has been shown to positively impact the preservation of nucleic acid quality within the sample [24] and to enhance DNA yields [25]. It remains unclear whether the incorporation of lysis tubes into the extraction process or sample homogenization within the DNA/RNA Shield contributes more significantly or equally to the enhanced DNA yields from processed hides. Further investigations are needed to elucidate whether comparable outcomes can be achieved solely by employing sample preservation in the DNA/RNA Shield and chemical lysis of the sample.

To ensure the normalization of the input amount of biological material during DNA extraction, two skin punch biopsy samples were obtained from the hide pieces using punch pliers. Despite the limited quantity of material, it was feasible to extract an adequate DNA amount from a single skin punch. Building upon our discoveries, we propose the collection of samples from various locations on tanned hides. This approach serves two purposes: first, to detect potential instances of false taxidermy and, second, to minimize the possibility of false-negative results in subsequent downstream analyses.

The findings from the qPCR quantification demonstrated a clear pattern of DNA degradation during the tanning process, as evidenced by a decrease in DNA yields and an increase in the rate of DNA fragmentation. This trend was particularly pronounced in the hides treated with the chromium sulfate tanning agent. Notably, intriguing results were obtained for the measured DNA concentrations in samples L3, L8-L, L8-N, L9-L, and L9-N. The minor variations observed in the measured DNA concentrations can be attributed to the fact that, despite efforts to standardize the collection of material for DNA extraction, it cannot be guaranteed that the skin punches used will be entirely identical in terms of the amount of input biological material.

A surprising decrease in DNA fragmentation was observed in sample L3 compared to samples L1 and L2. This unexpected result appears to be illogical since L3 is a sample derived from the same hide but taken at a different tanning phase, with the phases following a chronological sequence. In our methodology, the L1 phase represents the initial stage, and at the end of each subsequent phase, a representative sample was collected for further analysis. Consequently, it was assumed that the DNA concentrations would decrease and that the degree of DNA fragmentation would increase across the tanning phases. Therefore, if DNA fragmentation reaches a specific value in the L1 and L2 phases, it should either remain the same or be higher in the L3 phase.

One potential explanation for this unexpected observation could be attributed to the first chemical treatment of the hide that occurred in the L3 phase (pickling I). It is plausible that, after the L1 and L2 samples were taken, the DNA in those samples underwent further degradation (e.g., by microorganisms), while the DNA in the L3 sample remained relatively “preserved”, resulting in higher DNA yields and lower DNA fragmentation.

A similar situation was observed in the samples treated with Lutan FN and Novaltan AL, particularly noticeable in the quantification of the 133 bp amplicon in samples L8-L, L8-N, L9-L, and L9-N. Three potential explanations are proposed to account for this observation. The first possibility is an improved availability of DNA in the final stages of tanning. In those stages, the hide undergoes treatments such as oiling and tumbling, which lead to the softening of the hide. This could, in theory, release some of the DNA that has been crosslinked to collagen fibers. Another plausible explanation is contamination with foreign DNA. However, the argument against contamination is supported by the absence of similar results in the samples treated with the chromium sulfate tanning agent. Additionally, during all laboratory procedures, a strict set of rules preventing such contamination was followed. Thus, contamination may be ruled out as an explanation. The most plausible explanation is the overestimation of the 133 bp amplicon concentration in samples L8-L, L8-N, L9-L, and L9-N, which is attributed to the higher likelihood of amplifying the shorter amplicon than the longer amplicon in highly degraded DNA [26]. However, this explanation only accounts for the significant fluctuations in measured DNA concentrations during qPCR. The same trend was observed in the fluorometric measurements using the Qubit system (Figure 4, Figure 5 and Figure 6), where only the genomic DNA isolate was utilized, without any amplification. Fluorometric quantification detects the concentration of total double-stranded DNA in the sample through a fluorescent dye that specifically binds to DNA. The Qubit system therefore enables the quantification of all DNA present in a sample, encompassing nuclear DNA; mtDNA; and potentially DNA from various sources, e.g., microorganisms. Consequently, it is challenging to determine with certainty whether the measured DNA concentration solely represents leopard DNA. The consistent trend in both the qPCR and fluorometric quantification results suggests that this phenomenon occurred before or during DNA extraction. However, none of the presented explanations fully elucidate this phenomenon. Consequently, the cause of this phenomenon remains unclear and necessitates further investigation.

However, our results indicate that enough amplifiable DNA can be obtained from tanned hide samples treated with aluminum-based reagents.

The results of the DNA quantification in the samples from hide pieces tanned with the chromium sulfate tanning agent are consistent with the established hypothesis. The quantification of DNA in this sample was only possible by qPCR using a 133 bp amplicon; the amplification of the longer fragment yielded no significant results. This suggests that only DNA fragments that are <369 bp long are likely present.

A genetic analysis, in addition to determining species identity, plays a crucial role in individual identification through DNA profiling using STR genotyping. The demand for developing STR multiplexes for the individual identification of endangered species is on the rise. While existing STR multiplexes for the *Pantherinae* subfamily primarily focus on *Panthera tigris* identification [9,27], the recently published Ptig STRPlex [9] is also applicable for the DNA profiling of *Panthera pardus*.

To assess the DNA profiles, samples L1 and L9-L, L9-N, and L9-C representing the untanned hide and fully tanned hides, respectively, were selected. Complete DNA profiles were obtained for all tested samples, except for sample L9-C. However, working with highly degraded DNA poses challenges during DNA profiling due to the uneven amplification of fragments, increasing the risk of occurrence of technical artifacts, such as the pull-up effect (observed in sample L9-L) or incomplete DNA profiles due to allelic drop-out (seen in sample L9-C). One possible approach to mitigate this issue is to modify the primer design and reduce the amplicon size [27]. Comparing the obtained DNA profiles, a decrease in signal intensity was observed in the samples from the later stages of tanning, indicating an escalating rate of DNA degradation known as the “ski slope effect” [28,29].

Furthermore, the presence of microorganisms in the tanned hide samples may have contributed to the accelerated degradation of DNA.

Based on the conducted analyses, it was observed that the chromium sulfate tanning of the hides resulted in the most significant DNA degradation. We found that obtaining amplifiable DNA from such tanned hides for subsequent forensic genetic analyses is not always possible. To address this issue, the elemental composition of the tanned hide samples was examined to determine whether chromium was detectable in the hides before initiating genetic analyses. The X-ray fluorescence (XRF) method was employed for this purpose. Many subsequent downstream analyses used in wildlife forensic genetics (e.g., DNA typing or species determination using Sanger sequencing) rely on a certain amount of amplifiable DNA. These analyses are very costly and time-consuming; therefore, the detection of chromium in tanned hide before conducting genetic analyses could help to save laboratory resources that would otherwise be futile to use.

The XRF method was chosen for the detection of chromium and other elements in the tanned leather because it is a rapid and non-invasive method for the determination of the elemental composition. As this method does not consume the material being analyzed, it is very suitable for use in forensic samples [30].

In conclusion, although every effort was made to preserve the integrity of the tested samples as much as possible, all the samples contained DNA in a highly degraded state. Placing samples in the DNA/RNA Shield immediately after removal from a given tanning step could significantly slow the process of DNA degradation in the samples after collection. However, for real forensic samples, the rate of DNA degradation can be expected to be even higher, considering that they can contain “aged DNA” if tanned in a period significantly prior to the analysis. Therefore, it is necessary to perform the same analyses on real forensic samples.

All the findings presented in this study are only applicable to the selected tanning methods. For a comprehensive study of the issue, the same analyses would need to be performed on a larger number of samples of leather tanned using other methods. However, the fulfillment of such an undertaking is limited by the number of forensic samples provided for scientific purposes. In the laboratory, attempts can be made to simulate conditions reflecting the nature of real forensic samples as best as possible, but it is problematic to replicate, for example, the tanning process, as it is usually not known exactly how a given hide was tanned.

Since we have found that further testing is necessary, this work can be considered a pilot study of the problem.

## 5. Conclusions

This pilot study is the first to analyze the influence of the chemicals used in the process of hide tanning on DNA degradation. All used tanning agents had a negative impact on the degree of DNA fragmentation, with chromium sulfate having the most negative effect. However, the hides treated with aluminum-based tanning agents yielded enough amplifiable DNA for subsequent downstream analyses. 

Based on the analyses conducted, we propose the following standard operating procedure for the genetic analysis of processed hides:

- The determination of the elemental composition of the tanned dermal tissue (e.g., via the XRF method) and the quantification of the chromium content.
- The acquisition of biological samples (via punch biopsy) from various distinct locations on the dermal tissue for DNA extraction.
- The utilization of the optimized extraction protocol outlined earlier for DNA extraction.
- The purification of the DNA isolates to eliminate copurified PCR inhibitors.
- The quantification of DNA utilizing quantitative PCR (qPCR) with two amplification targets, a shorter fragment (up to 150 bp) and a longer fragment (up to 400 bp), followed by the determination of the degradation index.
- An assessment of the quantification results and the selection of appropriate methodologies for subsequent analyses.

## Figures and Tables

**Table 1:** Description of the samples and the tanning stage from which they were obtained.

Sample Label	Tanning Stage
L1	Untanned skin
L2	Degreasing solution
L3	Pickling solution I
L4	Fleshing and second degreasing bath
L5	Pickling solution II
L6-L	Tanning solution—Lutan FN
L6-N	Tanning solution—Novaltan AL
L6-C	Tanning solution—chromium sulfate
L7-L	Tanning solution—Lutan FN with the addition of Eulan SPA 01
L7-N	Tanning solution—Novaltan AL with the addition of Eulan SPA 01
L7-C	Tanning solution—chromium sulfate with the addition of Eulan SPA 01
L8-L	Leather oiling (Lutan FN tanned skin)
L8-N	Leather oiling (Novaltan AL tanned skin)
L8-C	Leather oiling (chromium sulfate tanned skin)
L9-L	Tumbling—Milling (Lutan FN tanned skin)
L9-N	Tumbling—Milling (Novaltan AL tanned skin)
L9-C	Tumbling—Milling (chromium sulfate tanned skin)

**Table 2:** Composition of solutions used in the tanning process.

Solution	Composition
Degreasing solution I	water (30 °C) + Supralan 67 1 mL/L + Supralan 809 1 mL/L + disinfectant Sanitol 0.5 mL/L + deodorizer 0.25 mL/L + 9 g NaCl/L
Degreasing solution II	water (38 °C) + Supralan 67 2 mL/L + Supralan 809 3 mL/L + NaCl 80 g/L
Pickling solution I	water (room temperature) + NaCl 120 g/L + formic acid 8 g/L
Pickling solution II	water (room temperature) + NaCl 80 g/L + formic acid 6 g/L
Tanning solution—Lutan FN	water (room temperature) + NaCl 80 g/L + Lutan FN 8 g/L + Prinol M31 3 g/L + Pelgrassol SF 4 mL/L + Eulan SPA 01 2 mL/L
Tanning solution—Novaltan AL	water (room temperature) + NaCl 80 g/L + Novaltan Al 8 g/L + Prinol M31 3 g/L + Pelgrassol SF 4 mL/L + Eulan SPA 01 2 mL/L
Tanning solution—chromium sulphate	water (room temperature) + NaCl 80 g/L + chromium sulfate 12 g/L + Prinol M31 3 g/L + Pelgrassol SF 4 mL/L + Eulan SPA 01 2 mL/L

**Table 3:** Primers used for qPCR.

Primer	Sequence 5′ → 3′	Amplicon Length	Reference
28S-F	GTTGTTGCCATGGTAATCCTGCTCAGT	133 bp	[18]
28S-R	TCTGACTTAGAGGCGTTCAGTCATAAT		
CY-F	CAACCCCACCGTGTTCTTCG	369 bp	[19]
CY-R	TTGCCATCCAGCCACTCAGTC		

**Table 4:** Composition of qPCR Master Mix.

Master Mix	15 μL Reaction	Final Concentration
iQ SYBR Green Supermix	7.5 µL	/
Primer forward (10 µM)	0.3 µL	0.3 µM
Primer reverse (10 µM)	0.3 µL	0.3 µM
Template DNA	1 µL	different
H_2_O	5.9 µL	/

**Table 5:** qPCR amplification protocol.

qPCR Program			Melting Curve Analysis		
Step	Temperature	Time	Step	Temperature	Time
1	95 °C	3 min	1	95 °C	15 s
2	95 °C	15 s	2	60 °C	15 s
3	60 °C	1 min	3	95 °C	15 s
Number of cycles: 40					

**Table 6:** Calculated values of the degradation index.

Degradation Index			
Tanning Stage	Lutan FN	Novaltan AL	Chromium Sulphate
L1	6.8	6.8	6.8
L2	101.4	101.4	101.4
L3	38.1	38.1	38.1
L4	17.2	17.2	17.2
L5	32.7	32.7	32.7
L6	7004.3	2933.6	non-determinable
L7	10,564.2	5180.4	non-determinable
L8	66,268.1	3587.0	non-determinable
L9	2930.1	19,890.3	non-determinable

## Data Availability

The data presented in this study are available on request from the corresponding author. The data are not publicly available due to ongoing research that has not yet been published.

## Supplementary Materials

The following supporting information can be downloaded at https://www.mdpi.com/article/10.3390/life14010147/s1, Figure S1: DNA profile obtained from the L1 sample—Ptig STRPlex 1; Figure S2: DNA profile obtained from the L1 sample—Ptig STRPlex 2; Figure S3: DNA profile obtained from the L9-L sample—Ptig STRPlex 1; Figure S4: DNA profile obtained from the L9-L sample—Ptig STRPlex 1 detail; Figure S5: DNA profile obtained from the L9-L sample—Ptig STRPlex 2; Figure S6: DNA profile obtained from the L9-N sample—Ptig STRPlex 1; Figure S7: DNA profile obtained from the L9-N sample—Ptig STRPlex 2; Figure S8: DNA profile obtained from the L9-C sample—Ptig STRPlex 1.

## References

[1] Scheffers B.R., Oliveira B.R., Lamb I., Edwards D.P. (2019). Global wildlife across the tree of life. Science.

[2] Maxwell S.L., Fuller R.A., Brooks T.M., Watson J.E.M. (2016). Biodiversity: The Ravages of Guns, Nets and Bulldozers. Nature.

[3] CITES CITES Appendices I, II, and III. https://cites.org/eng/app/appendices.php.

[4] World Bank Group Analysis of International Funding to Tackle Illegal Wildlife Trade. https://documents.worldbank.org/en/publication/documents-reports/documentdetail/695451479221164739/analysis-of-international-funding-to-tackle-illegal-wildlife-trade.

[5] Smart U., Cihlar J.C., Budowle B. (2021). International Wildlife Trafficking: A Perspective on the Challenges and Potential Forensic Genetics Solutions. Forensic Sci. Int. Genet..

[6] UNODC World Wildlife Crime Report—Trafficing in Protected Species. https://www.unodc.org/unodc/en/data-and-analysis/wildlife.html.

[7] Khedkar G., Khedkar C., Prakash B., Khedkar A., Haymer D. (2019). DNA Barcode Based Identification of a Suspected Tiger Skin: A Case to Resolve Mimicry. Forensic Sci. Int. Rep..

[8] Vankova L., Vanek D. (2022). DNA-Based Identification of Big Cats and Traditional Chinese Medicine Artifacts in the Czech Republic. Forensic Sci. Int. Genet. Suppl. Ser..

[9] Vanek D., Ehler E., Vanková L. (2021). Technical Note: Development of DNA Quantitation and STR Typing Systems for Panthera Tigris Species Determination and Individual Identification in Forensic Casework. Eur. J. Environ. Sci..

[10] Rajani C.V., Patki H.S., Simanta P., Surjith K., Deepa P.M., Pradeep M. (2020). Histomorphological Differentiation of the Skin of Leopard *(Panthera pardus*), Leopard Cat (*Prionailurus bengalensis*), Bengal Tiger (*Panthera tigris*), and Golden Jackal (*Canis aureus*). Vet. World.

[11] Votrubova J., Rihova P., Saskova L., Vanek D. (2017). Operation Tiger’s Eye: DNA Testing of Traditional Chinese Medicine Artifacts in the Czech Republic. Forensic Sci. Int. Genet. Suppl. Ser..

[12] Kite M., Thompson R. (2011). Conservation of Leather and Related Materials.

[13] Koehler G., Hobson K.A. (2018). Effects of Tanning on the Stable Isotopic Compositions of Hair. Forensic Sci. Int..

[14] Dubey R., Verma P., Kumar S. (2022). Cr (III) Genotoxicity and Oxidative Stress: An Occupational Health Risk for Leather Tannery Workers of South Asian Developing Countries. Toxicol. Ind. Health.

[15] Gouda S., Kerry R.G., Das A., Chauhan N.S. (2020). Wildlife Forensics: A Boon for Species Identification and Conservation Implications. Forensic Sci. Int..

[16] Vuissoz A., Worobey M., Odegaard N., Bunce M., Machado C.A., Lynnerup N., Peacock E.E., Gilbert M.T.P. (2007). The Survival of PCR-Amplifiable DNA in Cow Leather. J. Archaeol. Sci..

[17] Ražić S.E., Kopjar N., Kašuba V., Skenderi Z., Akalović J., Hrenović J. (2022). Evaluation of DNA-Damaging Effects Induced by Different Tanning Agents Used in the Processing of Natural Leather—Pilot Study on HepG2 Cell Line. Molecules.

[18] Hoffmann O.I., Kerekes A., Lipták N., Hiripi L., Bodo S., Szaloki G., Klein S., Ivics Z., Kues W.A., Bosze Z. (2016). Transposon-Based Reporter Marking Provides Functional Evidence for Intercellular Bridges in the Male Germline of Rabbits. PLoS ONE.

[19] Dawoud Al-Bader M., Ali Al-Sarraf H. (2005). Housekeeping Gene Expression during Fetal Brain Development in the Rat—Validation by Semi-Quantitative RT-PCR. Dev. Brain Res..

[20] Vernarecci S., Ottaviani E., Agostino A., Mei E., Calandro L., Montagna P. (2015). Quantifiler® Trio Kit and Forensic Samples Management: A Matter of Degradation. Forensic Sci. Int. Genet..

[21] Hedmark E., Ellegren H. (2005). Microsatellite Genotyping of DNA Isolated from Claws Left on Tanned Carnivore Hides. Int. J. Legal. Med..

[22] Onem E., Yorgancioglu A., Karavana H.A., Yilmaz O. (2017). Comparison of Different Tanning Agents on the Stabilization of Collagen via Differential Scanning Calorimetry. J. Therm. Anal. Calorim..

[23] Gould E.M., Taylor M.A., Holmes S.J. (2011). A More Consistent Method for Extracting and Amplifying DNA from Bee Wings. Apidologie.

[24] Vider J., Croaker A., Cox A.J., Raymond E., Rogers R., Adamson S., Doyle M., O’Brien B., Cripps A.W., West N.P. (2020). Comparison of Skin Biopsy Sample Processing and Storage Methods on High Dimensional Immune Gene Expression Using the Nanostring NCounter System. Diagn. Pathol..

[25] Trivedi C.B., Keuschnig C., Larose C., Rissi D.V., Mourot R., Bradley J.A., Winkel M., Benning L.G. (2022). DNA/RNA Preservation in Glacial Snow and Ice Samples. Front. Microbiol..

[26] Gill P., Bleka Ø., Fonneløp A.E. (2022). Limitations of QPCR to Estimate DNA Quantity: An RFU Method to Facilitate Inter-Laboratory Comparisons for Activity Level, and General Applicability. Forensic Sci. Int. Genet..

[27] Zou Z.-T., Uphyrkina O.V., Fomenko P., Luo S.-J. (2015). The Development and Application of a Multiplex Short Tandem Repeat (STR) System for Identifying Subspecies, Individuals and Sex in Tigers. Integr. Zool..

[28] Bright J.-A., Taylor D., Curran J.M., Buckleton J.S. (2013). Degradation of Forensic DNA Profiles. Aust. J. Forensic Sci..

[29] Schulze Johann K., Bauer H., Wiegand P., Pfeiffer H., Vennemann M. (2022). Detecting DNA Damage in Stored Blood Samples. Forensic Sci. Med. Pathol..

[30] Pouliot B.P., Mass J., Kaplan L. Using Xrf for the Identification of Chrome Tanning in Leather. Proceedings of the 43rd Annual Meeting in Miami.

