# Supplementary material for: The Influence of Tanning Chemical Agents on DNA Degradation: A Robust Procedure for the Analysis of Tanned Animal Hide—A Pilot Study
Source: Life (Basel). 2024 Jan 19;14(1):147. doi: 10.3390/life14010147 (PMC10817434; doi:10.3390/life14010147)
Supplement: Supplementary file 1 [file life-14-00147-s001.zip › life-2775290-supplementary.pdf]

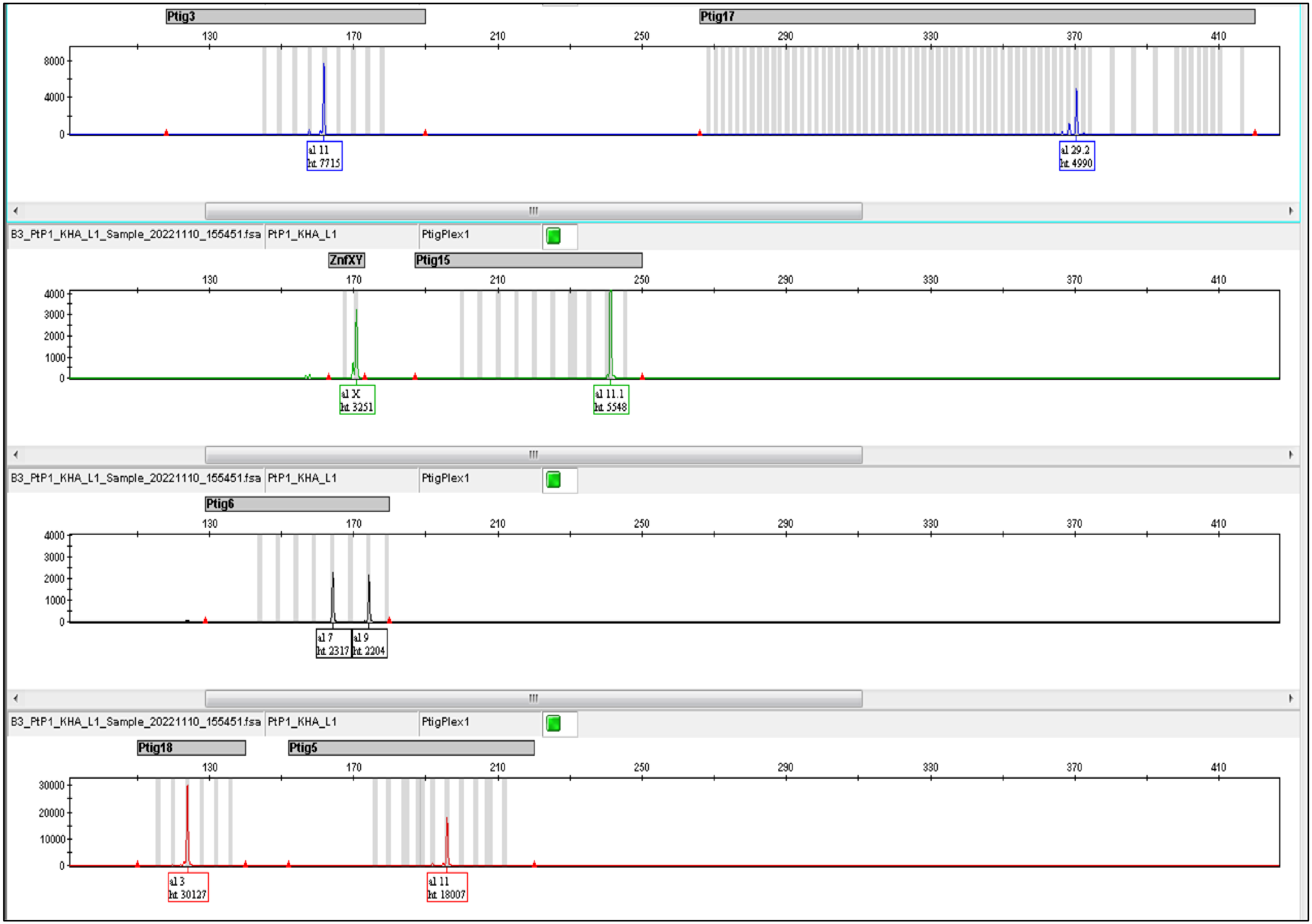

Figure S1. DNA profile obtained from the L1 sample – Ptig STRPlex 1.

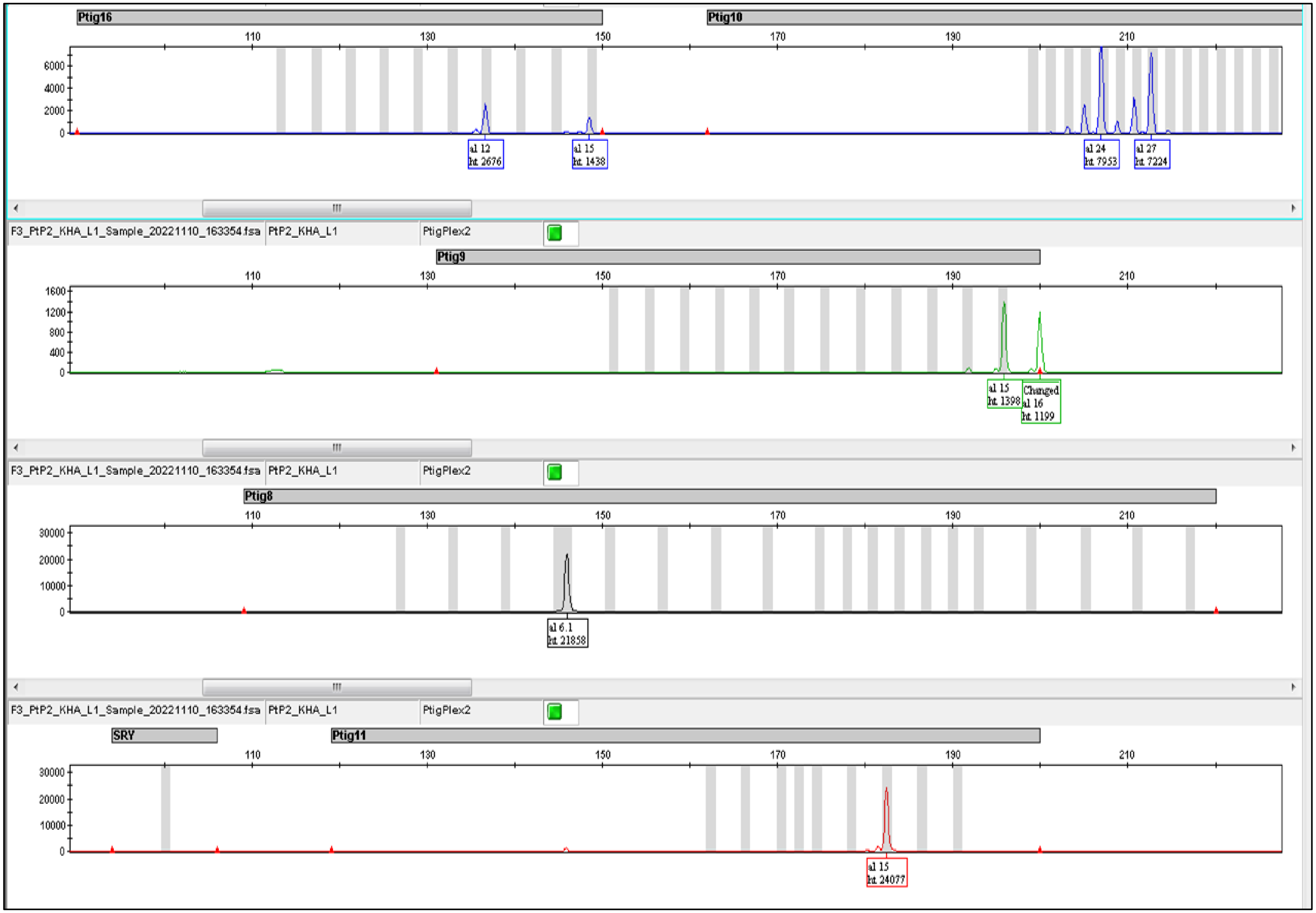

Figure S2. DNA profile obtained from the L1 sample – Ptig STRPlex 2.

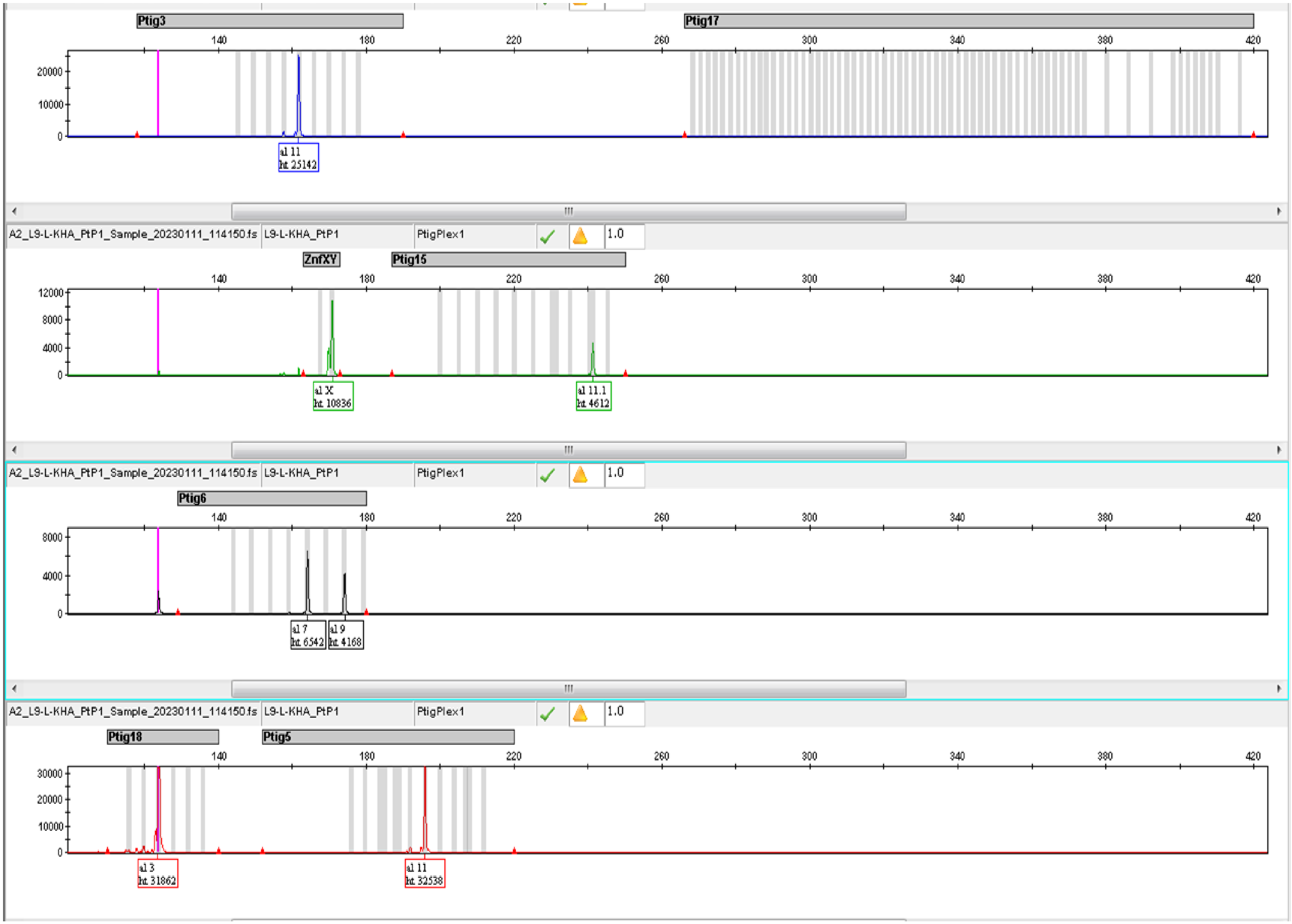

Figure S3. DNA profile obtained from the L9-L sample – Ptig STRPlex 1.

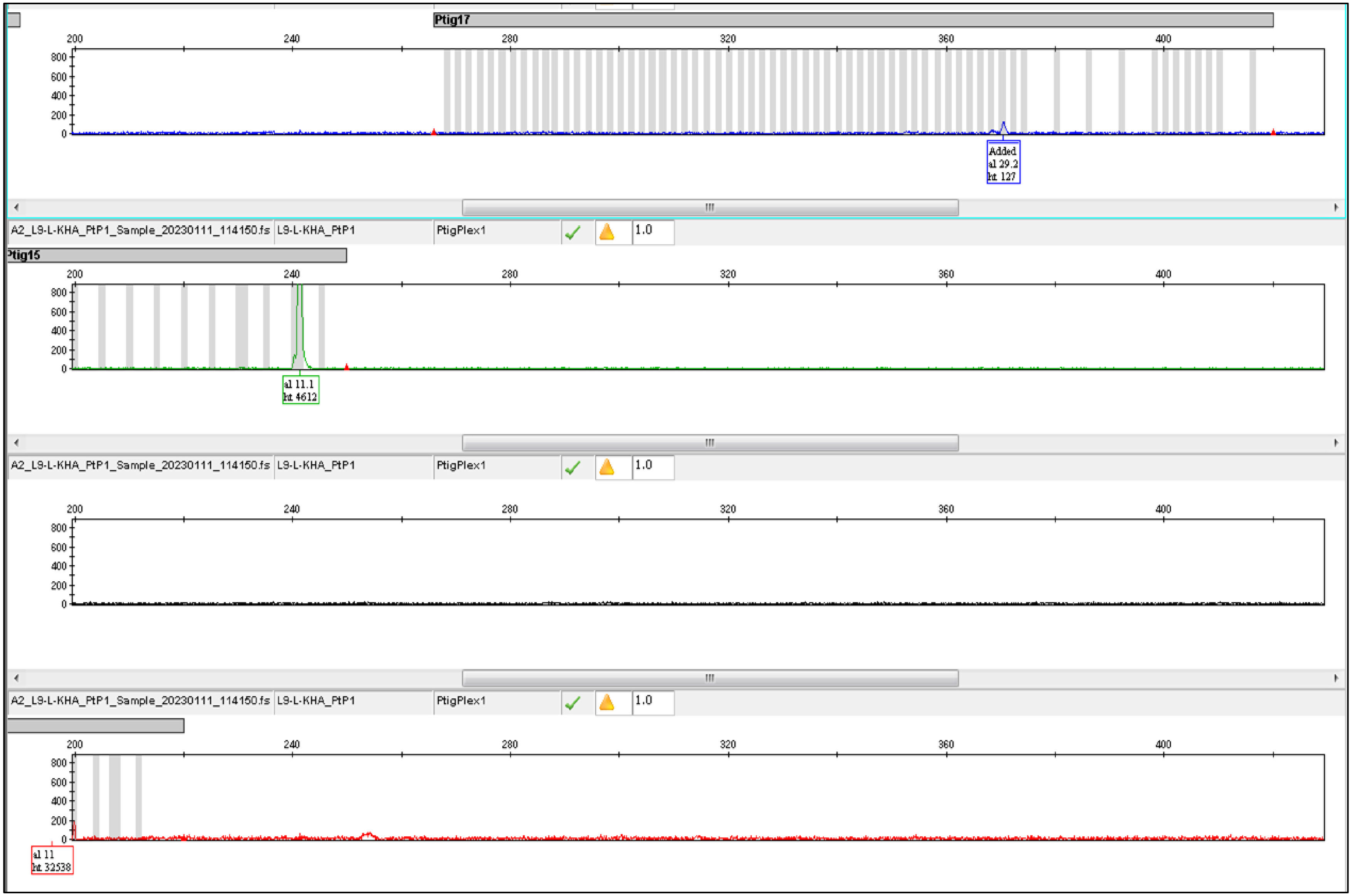

Figure S4. DNA profile obtained from the L9-L sample – Ptig STRPlex 1 detail.

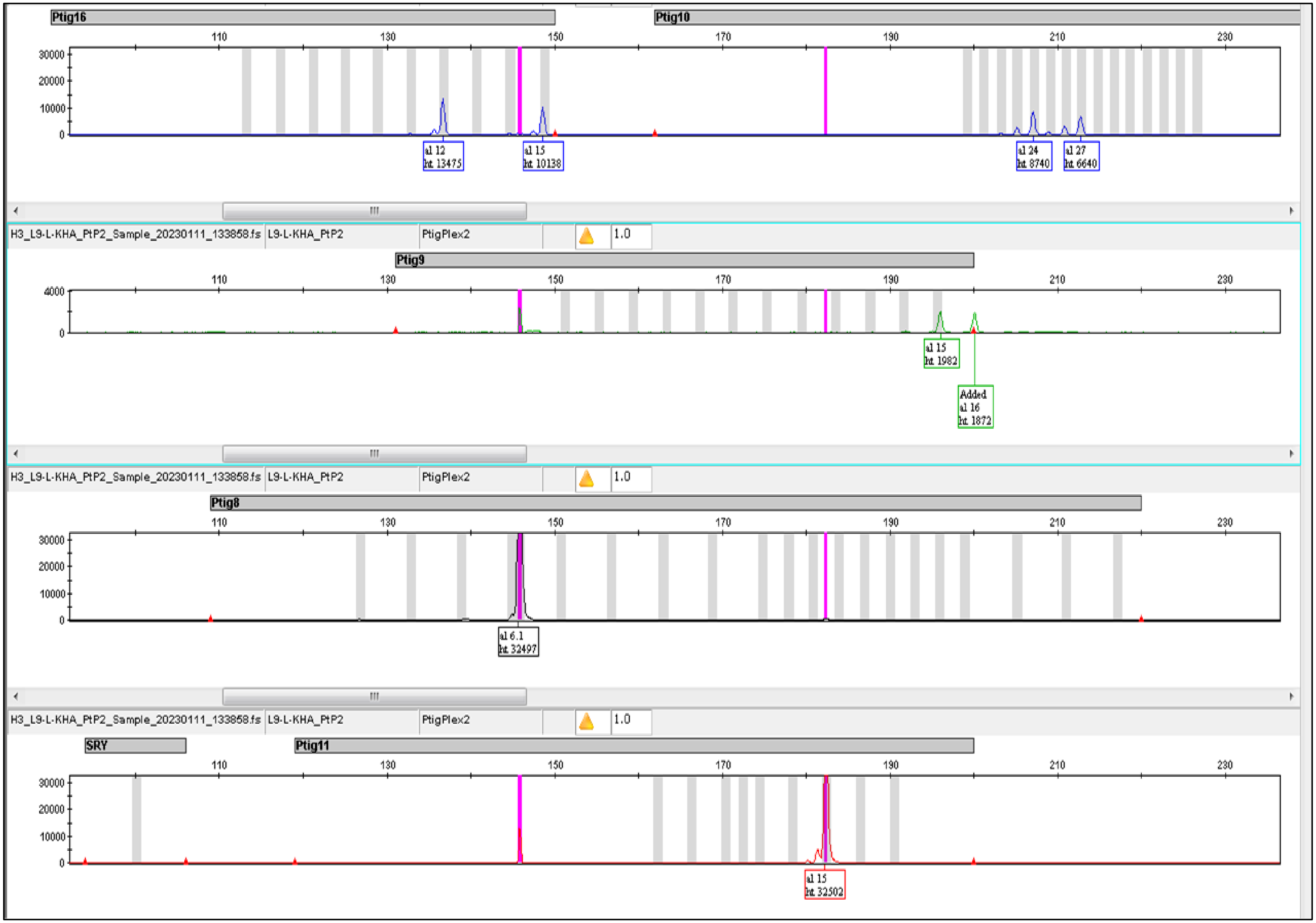

Figure S5. DNA profile obtained from the L9-L sample – Ptig STRPlex 2.

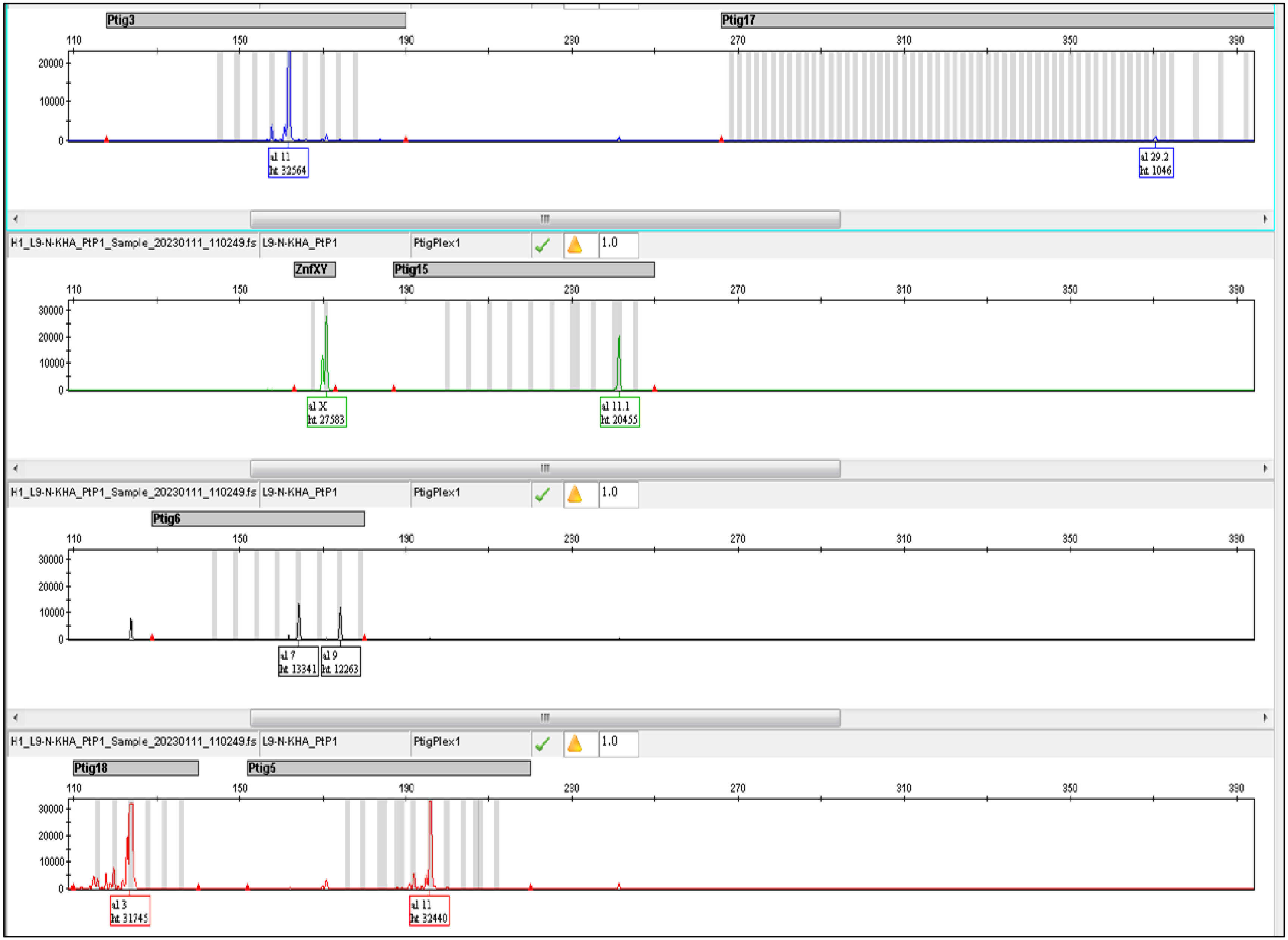

Figure S6. DNA profile obtained from the L9-N sample – Ptig STRPlex 1.

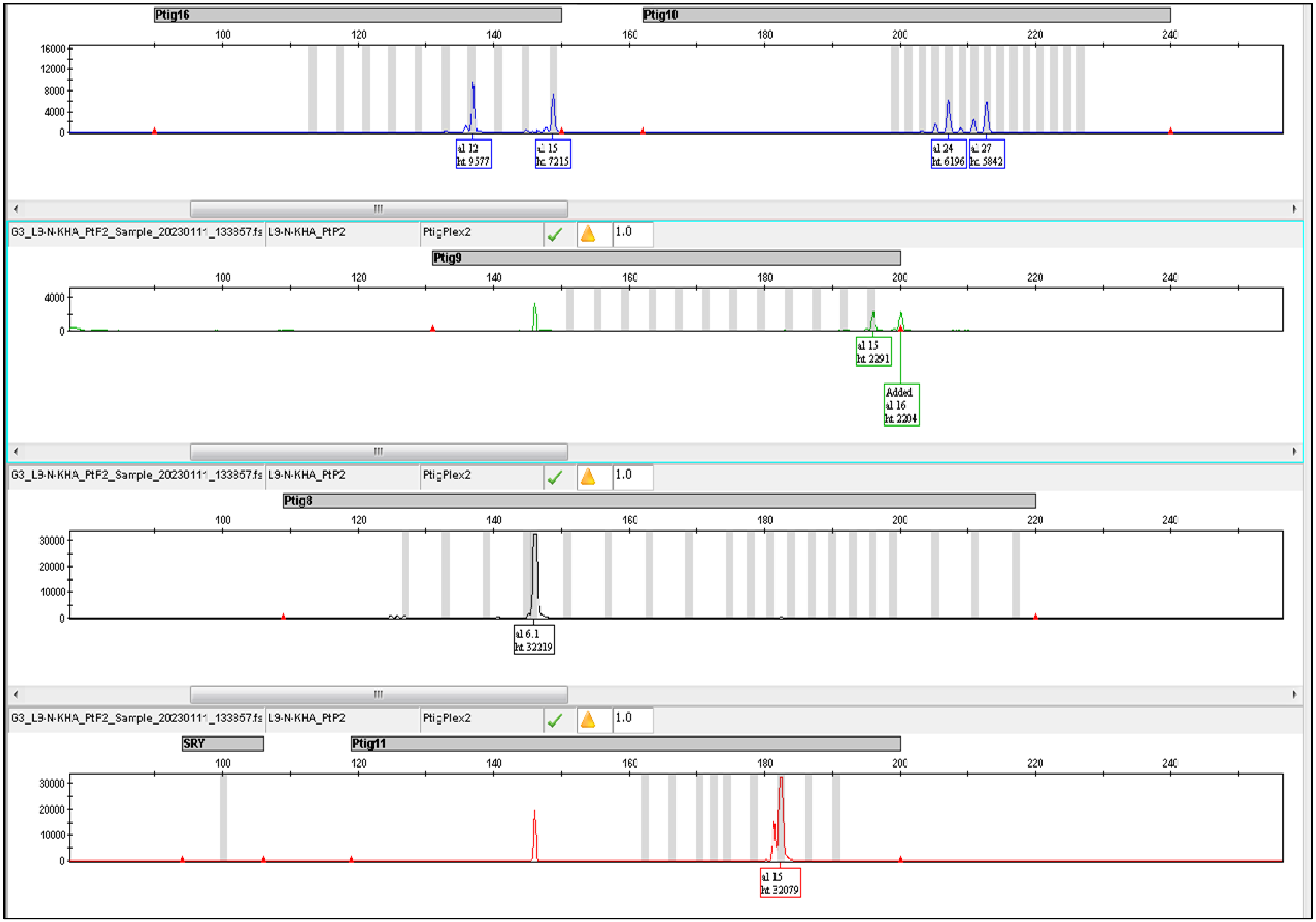

Figure S7. DNA profile obtained from the L9-N sample – Ptig STRPlex 2.

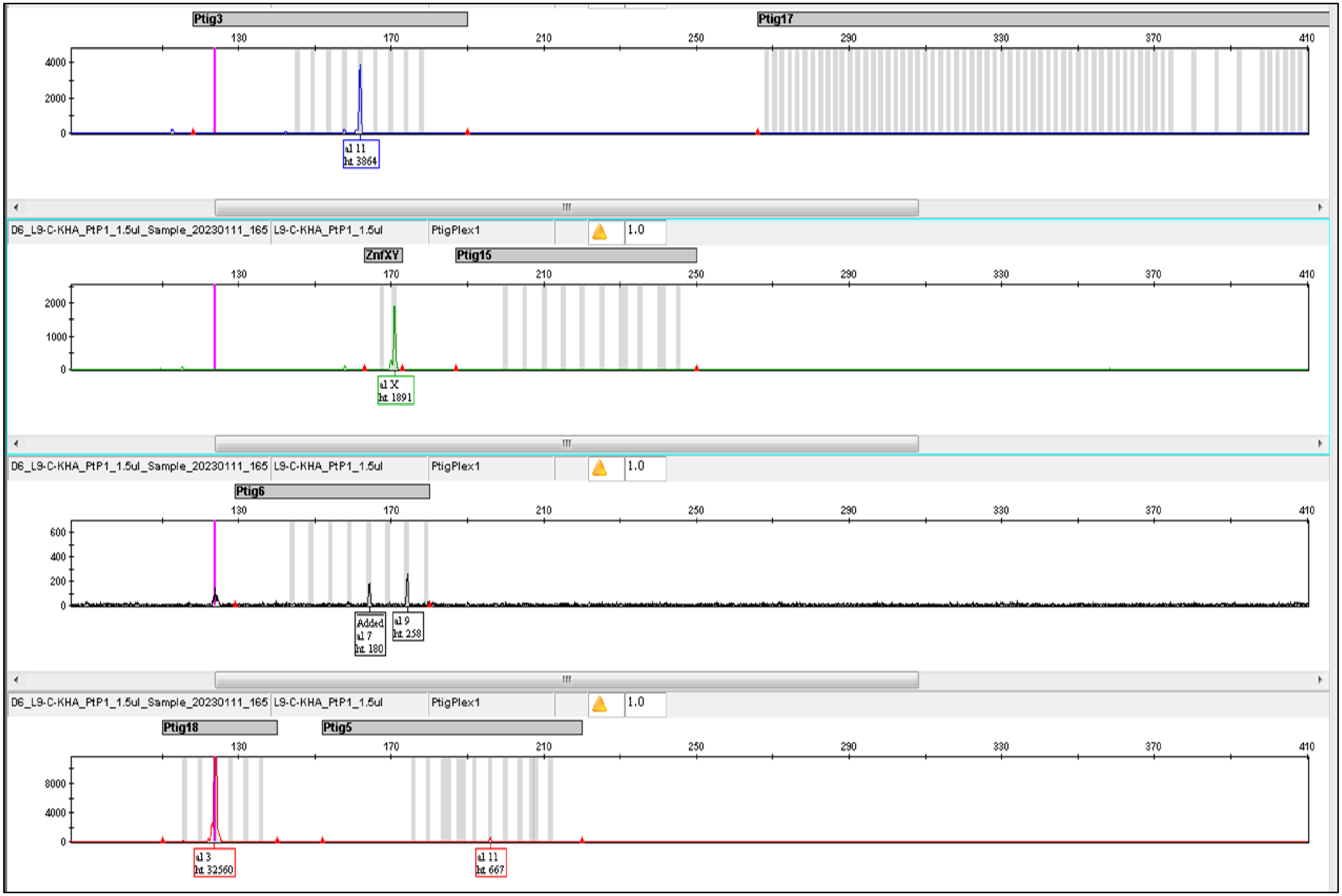

Figure S8. DNA profile obtained from the L9-C sample – Ptig STRPlex 1.
